# Anogenital distance in human male and female newborns: a descriptive, cross-sectional study

**DOI:** 10.1186/1476-069X-3-8

**Published:** 2004-09-13

**Authors:** Eduardo Salazar-Martinez, Patricia Romano-Riquer, Edith Yanez-Marquez, Matthew P Longnecker, Mauricio Hernandez-Avila

**Affiliations:** 1 National Institute of Public Health, Av. Universidad 655, Col. Santa Ma. Ahuacatitlan, 62508 Cuernavaca, Morelos, Mexico; 2 Mexican Institute of Social Security, Boulevard Benito Juarez #18 Tercer piso Col. Centro, C.P. 62000, Cuernavaca, Morelos, Mexico; 3 National Institute of Environmental Health Sciences, National Institutes of Health, Department of Health and Human Services, MD A3-05, PO Box 12233, Research Triangle Park, North Carolina 27709, USA

## Abstract

**Background:**

In animal studies of the effects of hormonally active agents, measurement of anogenital distance (AGD) is now routine, and serves as a bioassay of fetal androgen action. Although measurement of AGD in humans has been discussed in the literature, to our knowledge it has been measured formally in only two descriptive studies of females. Because AGD has been an easy-to-measure, sensitive outcome in animals studies, we developed and implemented an anthropometric protocol for measurement of AGD in human males as well as females.

**Methods:**

We first evaluated the reliability of the AGD measures in 20 subjects. Then measurements were taken on an additional 87 newborns (42 females, 45 males). All subjects were from Morelos, Mexico.

**Results:**

The reliability (Pearson r) of the AGD measure was, for females 0.50, and for males, 0.64. The between-subject variation in AGD, however, was much greater than the variation due to measurement error. The AGD measure was about two-fold greater in males (mean, 22 mm) than in females (mean, 11 mm), and there was little overlap in the distributions for males and females.

**Conclusion:**

The sexual dimorphism of AGD in humans comprises prima facie evidence that this outcome may respond to *in utero *exposure to hormonally active agents.

## Background

In animal studies of the effects of hormonally active agents, measurement of anogenital distance (AGD) is now routine [1-16], and serves as a bioassay of fetal androgen action. In rodents, perineal growth is dihydrotestosterone-dependent [17], males have a greater AGD than females, and use of AGD to sex newborns is standard [18]. In animals AGD is correlated at only modest levels with body weight [19], because these measures reflect the effects of endocrine axes that are largely independent. AGD usually tracks through life, varies by dose of antiandrogen, and can be predictive of other androgen-responsive outcomes [20].

Although measurement of AGD in humans has been discussed in the literature [19,21-23], to our knowledge it has been measured formally in only two descriptive studies of females [24,25]. Because AGD has been an easy-to-measure, sensitive outcome in animal studies, we developed and implemented an anthropometric protocol for measurement of AGD in human males as well as females. This work constitutes a modest step towards evaluation of AGD in human males as a potentially useful anthropometric measure and indicator of *in utero *androgen status.

## Methods

### Subjects

A cross-sectional study was conducted among the newborn children of women admitted for delivery to the Dr. Ernesto Meana San Román General Hospital in Jojutla, Morelos, Mexico, in 1999. This hospital provides medical care to low socioeconomic status and uninsured populations. The study included 87 newborn infants, none of whom had congenital defects or had been admitted to the neonatal intensive care unit. All infants were born at term (≥38 weeks gestation), except for one (32 weeks). The infants were of both sexes and were born after spontaneous cephalic delivery or caesarean section. Within 6 hours of birth, a structured questionnaire about family background and obstetric history was administered to the mothers, and anthropometric measurements were taken on the newborns.

### Anthropometry

**Figure 1:**
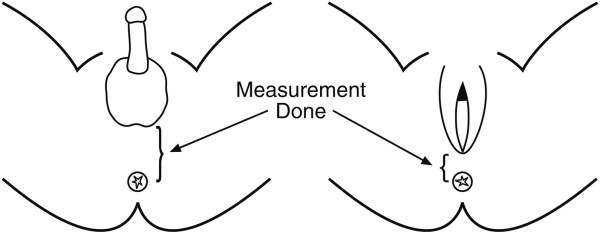
Schematic Diagram of Measurements Done, by Sex

Anthropometric measurements were taken of weight, length, head circumference, and AGD. AGD was measured as follows: the newborn infant was in the dorsal decubitus position; both hips were flexed and light pressure was exerted on the infant's thighs until the examiner's hand touched the subject's abdomen. Measurements were made with Vernier calipers. Distance was measured from the center of the anus to the posterior convergence of the fourchette (where the vestibule begins) in female infants [24]; and from the center of the anus to the junction of the smooth perineal skin with the rugated skin of the scrotum in male infants (Figure 1). Gestational age was estimated according to the Dubowitz scoring system [26].

### Reliability

Before any contact with the 87 subjects in the main study, the personnel performing the anthropometry examined 20 other neonates; all of whom were born after ≥38 weeks gestation. In this standardization training, 7 female infants and 13 male infants were measured twice by each observer. A sufficient time interval (30 minutes) was allotted between each measurement so that the second would not be influenced by the observer's memory of the first. These data were used to examine the reliability of measures and sources of variance.

### Statistical analysis

The reliability of the anthropometric measures was calculated as the Pearson correlation coefficient between the paired measures. The observations taken by the two observers were not statistically different when compared using a paired t-test (results not shown). Analysis of variance (ANOVA) with a random effect term for subject was used to estimate between-subject, between-observer, and within-observer components of variance, by sex.

For the main study, a linear regression analysis was used to evaluate birth weight, birth length, and gestational age as predictors of AGD. Age of the mother, number of pregnancies, and time elapsed between birth and measurement were not important predictors (or confounders) of AGD in univariate or multivariate models and were not considered further in the analysis. To examine influential values and the overall fit of the model, we conducted an analysis of residuals, but found nothing of note.

The protocol was approved by human subjects committees at the National Institute of Public Health in Mexico and the National Institute of Environmental Health Sciences in the U.S.

## Results

Among the 20 subjects in the standardization exercise, the between-subject coefficient of variation was greater for measures of AGD in females than for the other measures (Table 1). The reliability of the AGD measures were lower than for the traditional measures of anthropometry, with the female value being slightly lower than that for males. The variances estimated from the ANOVA were, for females: between-subjects, 7.9; between-observers, 0.6; and within-observer, 0.0. For males, the values were: between-subjects, 3.5; between-observers, 0.0; and within-observer, 0.1. The relative size of the variance components was unchanged when birth weight was included in the models. Thus, the between-subject variation in AGD was much greater than the variation due to measurement error.

**Table 1:** Mean, coefficient of variation (CV), and reliability of anthropometric measurements in 20 newborns^a^

Measurement	Mean	CV	Reliability
Weight (kg)	3.01	0.13	1.00
Length (cm)	48.9	0.03	0.97
Head Circumference (cm)	34.2	0.03	0.98
Anogenital distance	18	0.31	0.91
Female	11	0.27	0.50
Male	21	0.09	0.64

^a ^7 females and 13 males.

**Figure 2:**
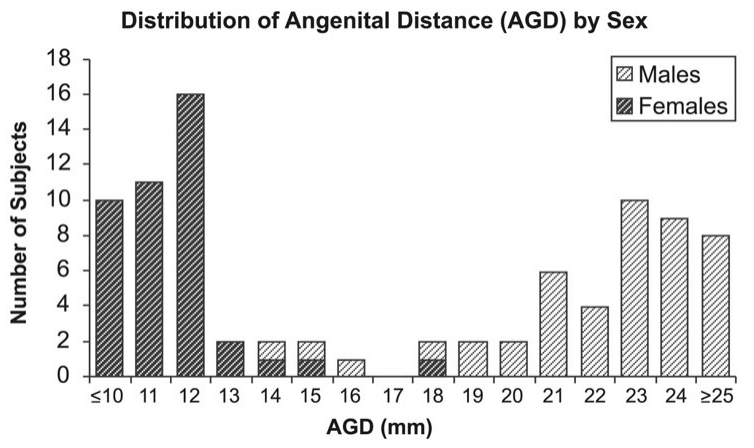
Distribution of Anogenital Distance (AGD), by Sex

Among the 87 subjects in the main study, the birth weight, length, and head circumference were as expected in a population from southern Mexico (Table 2) [27]. The AGD measure was about two-fold greater in males than in females, and there was little overlap in the distributions for males and females (Figure 2). The correlation of AGD with body weight was 0.64 in females and 0.48 in males.

**Table 2:** Distribution of selected characteristics in 87 newborns, Mexico, 1999^a^

Variable		Female n = 42	Male n = 45
Anogenital distance (mm)	Mean	11	21
	SD	2	3
	Median	11	22
	25^th ^percentile	10	20
	75^th ^percentile	11	23
Weight (g)	Mean	3070	3060
	SD	408	440
	Median	3060	3110
	25^th ^percentile	2870	2800
	75^th ^percentile	3310	3290
Length (cm)	Mean	48.6	48.7
	SD	1.4	2.2
	Median	48.6	48.7
	25^th ^percentile	47.5	48.0
	75^th ^percentile	49.6	49.9
Head circumference (cm)	Mean	337	341
	SD	10.9	16.7
	Median	337	341
	25^th ^percentile	330	334
	75^th ^percentile	345	350

^a^SD, standard deviation

In the crude models of AGD in females, weight, length, and gestational age all appeared to be predictive (Table 3). The adjusted results, however, suggested that weight of the newborn was the most important correlate, based on the p value being lower than for length or gestational age. For males, weight and length were more important than gestational age as determinants, and this pattern was seen also in the adjusted results (Table 4). Length had a slightly larger R^2 ^and slightly lower *p *value, suggesting it may be a marginally better predictor than weight in males. In a model of data for males that included weight, length, and gestational age, the *p *values for both length and gestation were less than 0.05, although the coefficient for gestation was negative. In a model of AGD based on data for males and females combined (results not shown), after adjustment for weight, the term for sex was clearly important (β for males = 10.9 mm, standard error = 0.4, *p *< 0.0001; change in R^2 ^due to addition of sex to model = 0.86).

**Table 3:** Regression coefficients for anogenital distance as a function of characteristics at birth, females^a^

Variable	Crude					Adjusted^b^				
		
	Coefficient	95%	CI	*p *value	R^2^	Coefficient	95%	CI	*p *value	R^2^
Birth weight	0.002	0.002	0.003	0.000	0.41	0.002^c^	0.001	0.003	0.000	0.43
Birth length	0.319	-0.005	0.642	0.061	0.09	0.141^c^	-0.189	0.471	0.407	0.22
Gestational age	1.296	0.516	2.076	0.002	0.21	0.501^d^	-0.282	1.283	0.217	0.43

^a^Units for regression coefficients are mm of AGD per unit characteristic (g, cm, or weeks). Results based on 42 females. CI, confidence interval.

^b ^Multivariate adjusted regression coefficients (adjustment factors listed below).

^c ^Adjusted for gestational age.

^d ^Adjusted for weight of newborn infant.

**Table 4:** Regression coefficients for anogenital distance as a function of characteristics at birth, males^a^

Variable	Crude					Adjusted^b^				
		
	Coefficient	95%	CI	*p *value	R^2^	Coefficient	95%	CI	*p *value	R^2^
Birth weight	0.003	0.001	0.005	0.001	0.23	0.004^c^	0.002	0.006	0.001	0.27
Birth length	0.671	0.348	0.995	0.000	0.28	0.914^c^	0.499	1.329	0.000	0.33
Gestational age	0.356	-0.258	0.971	0.262	0.03	-0.560^d^	-1.284	0.165	0.137	0.27

^a^Units for regression coefficients are mm of AGD per unit characteristic (g, cm, or weeks). Results based on 45 males. CI, confidence interval.

^b ^Multivariate adjusted regression coefficients (adjustment factors listed below).

^c ^Adjusted for gestational age.

^d ^Adjusted for weight of newborn infant.

## Discussion

The AGD measures employed in the present study reflect the location of the caudal border of the genital swelling, an embryologic structure that differentiates into the labia majora in females and the scrotum in males. After the indifferent stage of the external genitalia, the critical events determining the sexual dimorphism of AGD in humans begin when, relative to the anus, the genital swelling, urethral folds, and possibly the genital tubercle, move ventrally under the influence of androgens [28]. Elongation of the genital tubercle, which becomes the phallus, also occurs at this time. The difference between males and females in our data demonstrates sexual dimorphism of this particular measure of AGD. The two-fold difference in the aspect of AGD that we measured is not reflected in the schematic diagrams of human sexual differentiation we have seen [29,30], which is likely due to the previous lack of formal measures.

Direct comparison of our results with those in the two other studies with measures of anus-to-fourchette (AF) distance in female newborns [24,25] is hampered by different eligibility criteria, and possibly different ethnicities, in the three studies. For example, Callegari et al.'s subjects had a mean weight of 2,530 g; Phillips et al. did not present mean birth weight but subjects were required to have a birth weight above 2,750 g; and in our study the mean birth weight among females was 3,060 g. The mean AF distance in the Callegari et al. study was 10.9 mm; in the Phillips et al. study was 16.1 mm in Jews and 16.5 in Bedouins, and in the present study was 11 mm. Callegari reported no ethnic differences in their population (62.6% Hispanic, 28.7% black, and 8.7% white). Despite the ethnic-specific mean values noted above, Phillips et al. reported that Jewish females had a greater AF distance than did Bedouins. The similarity of the mean AF distance measures in the present study and the Callegari et al. study is surprising given the difference in mean birth weights, and suggests ethnic differences, or a systematic difference in how the measurements were done.

Compared with established anthropometric measures on newborns, the reliability of the AGD measures were lower. The lower reliability of the AGD measures is likely due to several factors. The AGD measures depend on indistinct landmarks on soft tissues. Structures such as "the center of the anus" or the posterior fourchette are not clearly demarcated. Any slight traction or pressure applied to the perineum or surrounding structures could alter measures. Finally, compared with established anthropometric measures on newborns, the AGD dimensions are smaller, thus measures done with the naked eye on a subject unlikely to hold still are inherently at a disadvantage. Use of two observers, one to restrain the subject and one to do the measurements could result in improved reliability compared to our approach, which employed one observer.

Compared with adult humans, the size of the genitals at birth is large relative to the body overall [28]. Yet the genital size is, of course, still determined in part by overall body dimensions and age. The need to adjust AGD for overall body dimension is well known in animal experiments [19]. In humans, the best approach to such adjustment remains unclear. Our data suggest that for the aspect of AGD we measured, adjustment for body weight is reasonable.

A complete assessment of AGD in humans would include more measurements than were done in our study. In neonatal rodents, measurement of AGD is relatively straightforward and is the distance from the genital tubercle to the anus. In older animals or humans of any age, however, questions arise as to which measure is most informative. For example, in human males, rather than a genital tubercle, the presence of the phallus and testicles at birth means that a number of measurements are possible. The measurement in the present study, from the posterior scrotal-perineal junction, represents only one such measurement. Ideally we would have done genital tubercle measurements in males and females, but we did not. Whether sexual dimorphism exists in the distance from the anus to the genital tubule (penile base in males) would be useful to know. While one might expect that penile length may be a good measure of androgenization among males, difficulties obtaining a reliable measure mean that alternative measures, such as AGD, are worth investigating.

Effects of endocrinopathies on AGD in humans have been described, but only to a limited degree. A rare form of congenital adrenal hyperplasia that causes incomplete masculine development has been reported to cause decreased AGD in boys [21]. Details on how the measurement was done (and the measured values), however, were not presented [22,23]. Callegari et al. [24] measured the distance from the anus to the fourchette (same as what we did) and in addition measured the distance from the anus to the clitoris; the ratio of these two measures in three newborn females with congenital adrenal hyperplasia was increased relative to normal newborn females. Earlier case reports on females with adrenogenital syndrome noted labiosacral fusion, but again, no formal measures were published [23]. The utility of AGD measures in humans is further supported by experimental data in primates showing that *in utero *exposure of females to androgenic agents increased AGD [1].

The purported mechanism by which androgens increase AGD in females is by inducing "labioscrotal fusion" (in normal males fusion begins caudally and proceeds ventrally, presumably androgens in females act the same way) [24]. This mechanism, however, does not account for why males who are not fully androgenized would have a decreased AGD, unless AGD in males is defined as being from tip of penis to the center of the anus. A set of formal AGD measures on subjects with selected congenital endocrinopathies or birth defects could be useful in evaluating whether this outcome is uniformly responsive to gross stimuli, and may help discern details of normal embryology and the consequences of disrupting it.

## Conclusions

In summary, we have shown that an aspect of genital dimension that reflects migration of the genital swelling is sexually dimorphic in humans. Whether this particular measure, or other measures of AGD in humans, has any utility as markers of exposure *in utero *to hormonally active agents remains to be seen.

## Abbreviations

AF: anus-fourchette

AGD: anogential distance

ANOVA: analysis of variance

CI: confidence interval

## Competing interests

None declared.

## Authors' contributions

ES participated in the design of the study, carried out the measurements, and wrote the first draft of the manuscript. PR participated in the study coordination and data management. EY carried out and coordinated the measurements. ML originated the idea that AGD measurements in human males may be useful, revised the manuscript, and analyzed the data. MH conceived of the study and participated in its design and coordination. All authors read and approved the final manuscript.

## Pre-publication history

The pre-publication history for this paper can be accessed here:


